# The Origin of Amerindians: A Case Study of Secluded Colombian Chimila, Wiwa, and Wayúu Ethnic Groups and Their Trans-Pacific Gene Flow

**DOI:** 10.3390/genes16030286

**Published:** 2025-02-27

**Authors:** Antonio Arnaiz-Villena, Tomás Lledo, Carlos Silvera-Redondo, Ignacio Juarez, Christian Vaquero-Yuste, José Manuel Martin-Villa, Fabio Suarez-Trujillo

**Affiliations:** 1 Department of Immunology, School of Medicine, University Complutense of Madrid, 28040 Madrid, Spain; 2 Department of Genetics, Universidad del Norte, Barranquilla 081007, Colombia

**Keywords:** HLA, Amerindians, Wiwa, Chimila, Wayúu, trans-Pacific contacts, America population, Afro-Americans, Europeans, first free African Americans, Barranquilla, San Basilio Palenque

## Abstract

Background/Objectives: The Human Leukocyte Antigen (HLA) system is composed of a set of genes that codify glycoproteins presenting antigenic proteins to clonotypic T cell receptors in order to start the immune response. Class I and Class II classical loci exhibit high allelic diversity; some of them (or their specific combinations that form haplotypes) are quasi-specific or highly frequent in certain populations and thus are useful for population genetic studies. In this study, an HLA genetic comparison of Chimila, Wayúu, Wiwa, and Barranquilla Colombian nonrelated healthy individuals was carried out together with other populations from all over the world to trace their genetic origin, obtain a virtual transplantation list, and inform future epidemiology studies. Methods: HLA-A, -B, -DRB1, and -DQB1 alleles were sequenced using the PCR-SSOP–Luminex method to analyze the HLA genetic profile of each individual. The data obtained were subsequently processed with standard software to obtain HLA alleles, haplotype frequencies, and genetic distances compared with data from global populations to generate relatedness dendrograms and carry out a correspondence analysis. Results: The results obtained place the Chimila, Wayúu, and Wiwa populations phylogenetically close to the other North and South Amerindian populations included in this study. Amerindians are genetically separated from the rest of the world’s populations. Chimila, Wayúu, and Wiwa present unique extended HLA haplotypes and specific alleles, such as HLA-B*48 or HLA-A*24:01, shared with Oceanian populations. Conclusions: These genetic results and anthropological data support prehistorical trans-Pacific (bidirectional) contacts that contributed to the settlement of America and also suggest that the effects of ancient European gene flow cannot be discarded.

## 1. Introduction

**Figure 1:**
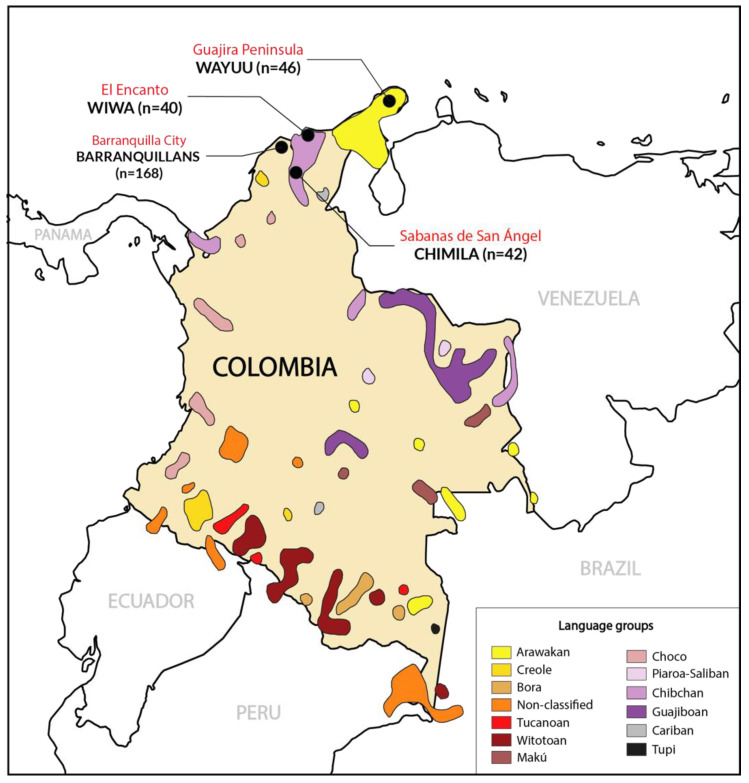
Map showing Colombia’s territory and the distribution of the main indigenous language groups currently spoken [7,8]. The map features the Guajira Peninsula, shared with Venezuela (Wayúu), El Encanto (Wiwa), Sabanas de San Ángel (Chimila; the latter 2 groups belong to the Santa Marta Snowed Sierra area), and urban Barranquilla city (Barranquillans). The locations where samples were taken are highlighted on themap.

Colombia is a country located in the northwestern region of South America that harbors a great cultural and linguistic diversity. Its population approximates 52 million inhabitants, of which about 4.4% corresponds to indigenous people [1]. At present, around 65 indigenous languages and 2 Creole languages are spoken in Colombia, which are distributed in 13 main different linguistic families (although this number depends on the author) [2,3] (Figure 1). Groups like Arawakan, Chibchan, and Chocó stand out because of their great number of speakers [3,4]. Many of the indigenous languages spoken today in Colombia are at serious risk of disappearing due to factors such as globalization, ethnic segregation, and lack of legislation for the cultural protection of languages. The Colombian government legislated for the preservation of linguistic diversity in the country in 2010, and various projects have been carried out for its conservation [5,6]. The present work is centered on the Amerindian ethnicities of northern Colombia. We considered three isolated populations, including Wayúu (Arawakan group), Chimila (Chibchan group), and Wiwa (Chibchan group) Amerindians, and also urban Barranquillan (“Barranquillero” in Spanish), in a neighboring admixed urban population as one of the outgroups, for a genetic study of their HLA profiles (Figure 1).

The Chimila, also known as Simiza, Shimizya, Chimile, or *eteenanaka* in their native language [9,10,11,12], are a people of Amerindian origin who currently live in the central plains of the Department of Magdalena and the Department of César, northernColombia [9,13,14]. In ancient times, the Chimila territory covered a large area from the Sierra Nevada de Santa Marta (Santa Marta Snowed Sierra) northeastern region in the North to the Ciénaga de Zapatosa in the South [9]. The Chimila inhabit extensive plains bathed by the Ariguaní River with a typically tropical climate that makes the region difficult to access. They have characteristic cultural features common to other Amerindian ethnic groups, such as turtle breeding, poison-arrow hunting, and the figure of the jaguar shaman [9,15]. The Chimila language (*etetaara*) [12,16,17,18] is usually included within the Chibchan linguistic group, although some authors expand this group to the Chibchan–Arawak group, shared with languages such as Kogi, Businka, or Bintúkua [11]. It is estimated that the current population of Chimila is around 1700 people [1].

The Wayúu (also called Guajiros) arean Amerindian ethnic group that inhabits the arid Guajira Peninsula in the northern Colombian and Venezuelan territories [9]. Around 97% of Wayúu people speak *wayuunaiki*, their native language [19,20]. They account for almost 400,000 people, according to the last population registry, although conducting a census is difficult due to the population spreading throughout the Guajira Peninsula [1]. They are organized into almost 30 non-exogamous matrilineal clans, and a “totem animal” is associated with each group, with Epieyú, Uriana, and Ipuana being the most numerous ones [9,21,22]. The Wayúu people have maintained their autonomy within the Colombian and Venezuelan territoriesand were recently constitutionally recognized by both countries. The *Wayuunaiki* language belongs to the Arawak family, with minimal dialectal differences among the Upper Guajira and Central–Bottom Guajira population groups [9,19,20].

The Wiwa, also called Sajas, Sanhas, Malayos, or Arsario [9,23], are a Colombian indigenous group that traditionally extends into the Department of El César, bordering the Department of La Guajira to the north [23]. They are also located in the Department of Magdalena territory, between the basins of the Guachaca, Jerez, and Tapias Rivers [23]. According to some authors, the most important and traditional Wiwa settlements in the Sierra Nevada de Santa Marta are *Kemakumake*, *Wimake,* and *Gotsezhi* (El Encanto) [23]. Currently, the Wiwa population is close to 20,000 individuals [1]; about half speak the ancestral language of the Wiwa, called *damana,* and are also classified into the Chibchan family [24,25]. They share the Sierra Nevada with the Kogi, Kankuama, and Ika (Arhuaco) ethnicities [9]; each of them speaks their own language, which alsobelongsto the Chibchan family [9]. The indigenous groups of the Santa Marta Sierra, including the Wiwa people, consider the delimitation of their territory based onthe *Linea Negra* (Black Line) [9,23], which includes the low and warm parts of the Sierra that contain numerous sacred places that they usually visit to performtheir rites.

Barranquilla city was officially founded by Spaniards in the year 1813 in a strategic place initially occupied by Camash Amerindians, and it is nowadays one of Colombia’s most important cities because it has become an important economic, cultural, and immigration center in the last centuries [26]. It is placed at the Magdalena River Mouth to the Caribbean Sea that connects northern and southern Colombia; its development has been closely linked to its port, which has been an economic engine for the northern region of Colombia since the 19th century, facilitating both international trade and cultural exchange. This river was widely used by Spaniards to introduce African slaves into South America, who sometimes fled and set up a “Palenque” or “free African city in America”, San Basilio de Palenque. These African citizens were the first free African Americans: they lived in America 100 years before Haitians were officially given freedom by the Spanish Crown [27,28,29,30]. African slaves were introduced through the Cartagena (Colombia) harbor, and most of them were carried to the Magdalena River to be finally transferred to Central and South Colombia and further through the Magdalena Riverbed [30,31].Thus, Baranquilla city emerged as an important cultural and economic center of Colombia after the Spanish colonization, attracting European, Arab, and Caribbean immigrants, which has generated a rich mix of influences on its demographics and culture. In modern times, Barranquilla is known for its carnival, recognized by UNESCO as an Intangible Cultural Heritage of Humanity [32], where the coexistence of African, indigenous, and European traditions is reflected. The variant of Spanish spoken in Barranquilla is called *españolbarranquillero*, being part of the Caribbean–Spanish dialects [33,34,35].

In the present work, we aim to analyze the HLA-A, -B, -DRB1, and -DQB1 profiles of the Chimila, Wayúu, Wiwa, and Barranquillan populations (the latter as a mixed outgroup) and to compare them with other Amerindian and worldwide populations in order to assess relatedness among them and evaluate the Amerindian genetic uniqueness and their possible transoceanic contacts with Pacific populations.

## 2. Materials and Methods

### 2.1. Population Samples

A total of 296 healthy, unrelated Colombian Amerindian individuals were included in this study, belonging to the following ethnicities: Wayúu (*n* = 46), Wiwa (*n* = 40), Chimila (*n* = 42), and Barranquillans (*n* = 168) (Figure 1). The Wayúu individuals were selected from an isolated population at the Guajira Peninsula (Guajira Department, Colombia). The Wiwa samples were collected from an isolated population at El Encanto (Sierra Nevada de Santa Marta, Northern Colombia). The Chimila participants were born at Sabanas de San Ángel (Department of Magdalena, Colombia), and the Barranquillan participants were selected from Barranquilla city, North Colombia. Every participant in this study volunteered to donate blood, and samples were collected after they signed consent at the Universidad del Norte, Colombia. The selected individuals spoke the native languages of their ethnic region (*ettetaara* for Chimila, *wayuunaiki* for Wayúu, *damana* for Wiwa, and *españolbarranquillero* for Barranquillans), and at least two generations back (grandparents), they were born in the isolated places mentioned above. The samples were used for HLA typing and phylogenetic calculations, together with other Amerindian and worldwide populations (see Table 1) [35]. This study was approved by the Complutense University of Madrid and Hospital Universitario 12 de Octubre de Madrid Ethical Committees. All subjects included in this study were adult unrelated blood donors who signed an informed consent form to participate.

**Table 1:** Populations used in the present study for HLA profile comparison. The first column includes Amerindians down to Mazatecans. The rest of the populations cover the worldwide range.

Population	*n*	Reference	Population	*n*	Reference
Chimila			Aleuts	104	[36]
Wayúu			Cape York	80	[37]
Wiwa			Kimberley	82	[37]
Barranquilla			Ainu	50	[38]
Mixteco	96	[39]	Yuendumu	119	[40]
Mayos	60	[41]	Papua New Guinea	57	[42]
Tarahumaras	44	[43]	New Caledonians	65	[42]
Terena	60	[44]	Rabaul	60	[42]
Lamas	83	[45]	Mandang	65	[42]
Lakota Sioux	302	[46]	Central-Desert	152	[40]
Nahuas	73	[47]	Fidji	57	[42]
Teenek	53	[48]	Western Samoa	102	[49]
Aymaras	87	[50]	Koreans	100	[51]
Quechuas	69	[52]	Buyi	70	[51]
Mayans	132	[53]	Chinese Singapore	71	[51]
Jaidukama	39	[54]	Tlinglit	53	[51]
Toba-Pilaga	19	[55]	Manchu	50	[51]
Arhuaco	123	[56]	Japanese	493	[51]
Zapotecans	75	[57]	Mongolians-Khoton	85	[58]
Mataco-Wichi	49	[55]	Mongolians-Khalk	202	[58]
Eastern Toba	135	[55]	Tuvinians	197	[59]
Xavantes	74	[55]	Chuvashians	82	[60]
Cayapa	100	[61]	Russians	200	[62]
Kogi	67	[56]	Germans	295	[51]
Arsario	20	[56]	Danish	124	[51]
Seri	100	[57]	Italians	284	[51]
Mixe	55	[57]	Spanish	176	[63]
Guarani	32	[57]	Spanish Basques	80	[63]
Mapuche	104	[64]	Algerians	102	[65]
Uros	105	[66]	Sardinians	91	[51]
Mazatecans	89	[67]	French	179	[51]
Athabaskans	124	[68]	Lebanese-NS	59	[69]
Evenks	35	[70]	Lebanese-KZ	93	[69]
Kets	22	[70]	Moroccan Jews	94	[71]
Udegeys	23	[70]	Berbers-Souss	98	[72]
Nivkhs	32	[70]	Cretans	124	[73]
Chukchi	59	[70]	Albanians	65	[51]
Eskimos	35	[70]	Macedonians	172	[74]
Koryaks	92	[70]	Moroccans	98	[75]

**Figure 2:**
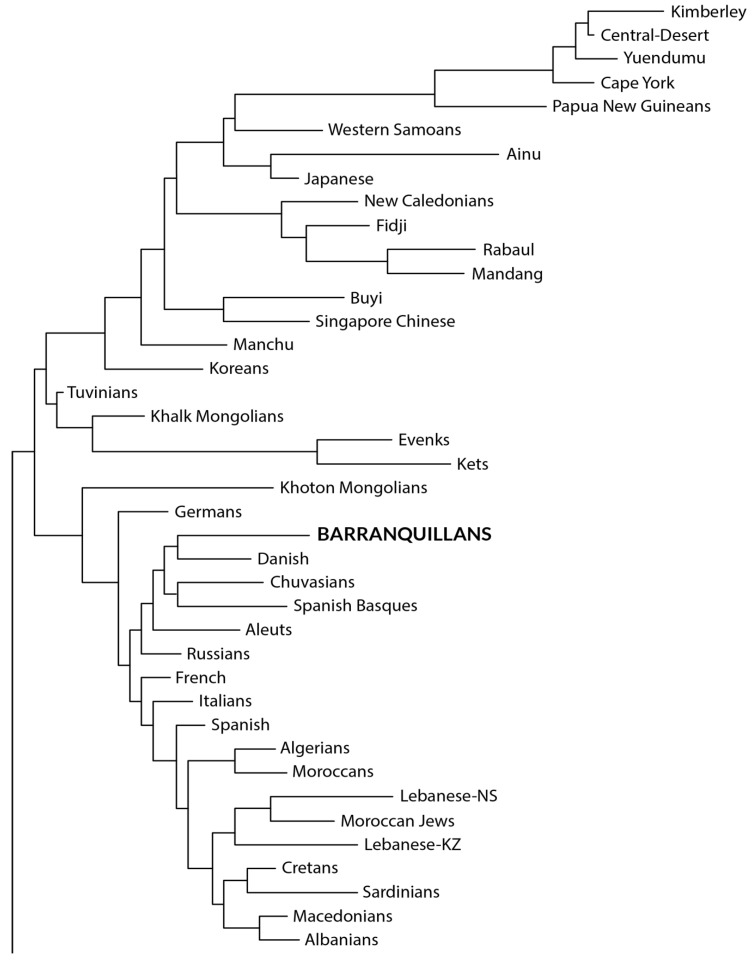

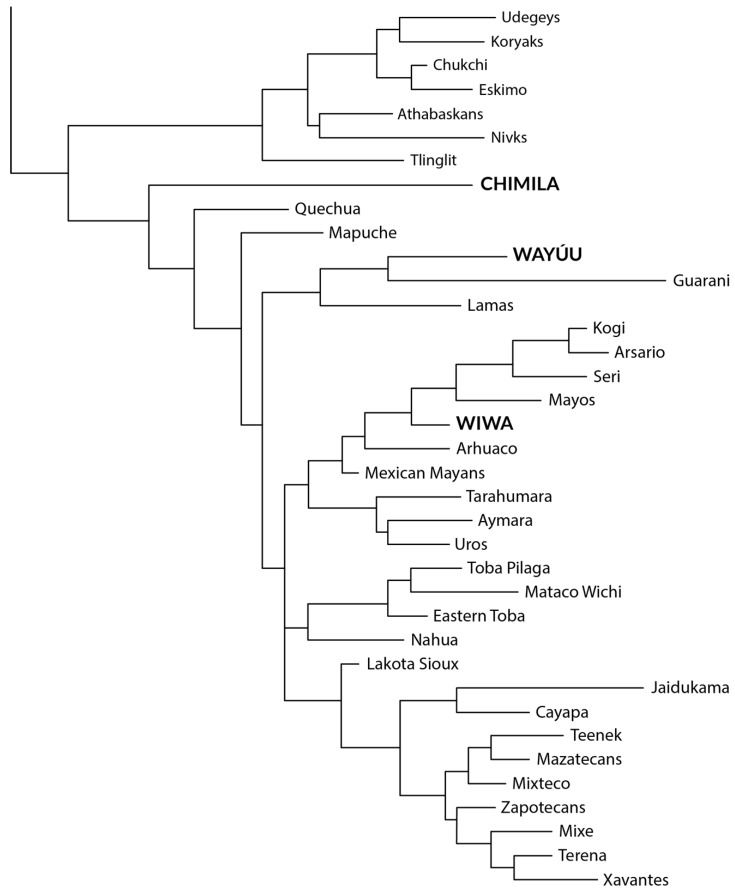
Neighbor-Joining relatedness dendrogram constructed by using genetic distances (DA). Amerindian populations (**bottom**) appear as a cluster separated from Na-Dene/Eskimo populations and located separate from Asiatics and Caucasians. In total, 100 bootstrap values are included in all nodes of the phylogenetic tree.

**Figure 3:**
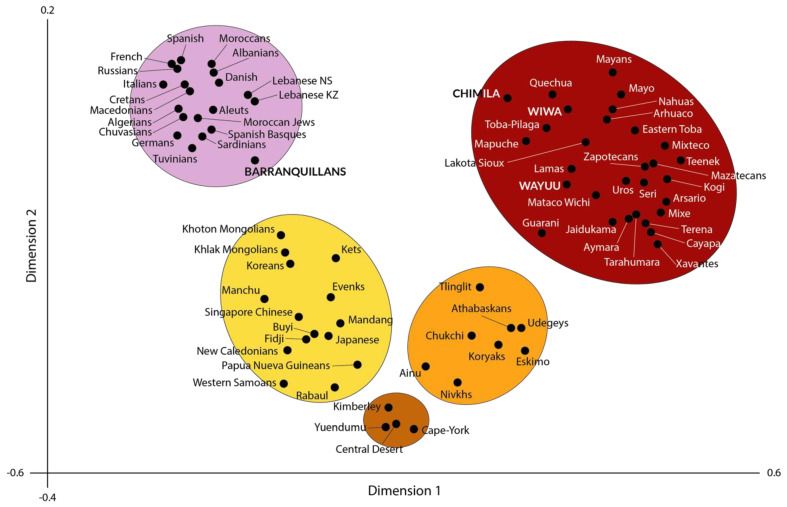
Correspondence analysis performed by using HLA-DRB1 frequencies from the high-resolution Chimila, Wiwa, Wayúu, and Barranquillan samples studied in the present work together with frequencies of other Amerindian and worldwide populations included for comparisons. Note that five different clusters were obtained in the analysis: Caucasians (**top left**, light purple), Asiatics (**bottom left**, yellow), Australian Aborigines (**bottom**, brown), Eskimo/Na-Dene (**bottom right**, orange), and Amerindians (**top right**, red).

Worldwide populations are listed and used for comparison with the Colombian Amerindian population and admixed Barranquillans; these comparisons are depicted in Figure 2 and Figure 3. The number of individuals is depicted in one column (*n*=). The article reference for each population studied is provided in brackets, from which data were taken to construct Figure 2 and Figure 3.

### 2.2. HLA Typing, DNA Sequencing, and Statistics

High- and low-resolution HLA class I (A and B) and HLA class II (DRB1 and DQB1) typing was carried out following a standard PCR-SSOP–Luminex protocol [76]. Some HLA class I typings in the studied populations were only available at low resolution after typing due to local typing limitations; thus, the HLA typings were homogenized at low resolution for a better visualization and comprehension of the data in some populations.

HLA-A, -B, -DRB1, and -DQB1 allele frequencies, Hardy–Weinberg equilibrium, and the linkage disequilibrium between studied loci calculations were performed with Arlequin v3.0 software [77]. The level of significance (*p*) for 2 × 2 comparisons was determined as previously described [51,78,79]. In addition, the most frequent extended haplotypes were deduced and only taken into account if they appeared in two or more individuals and the alternative haplotype was well-defined [51,78,79].

We subsequently compared our Chimila, Wayúu, Wiwa, and Barranquillan high-resolution HLA-DRB1 data obtained in present work with those of Pacific Islanders, Caucasian Europeans, Siberians, Orientals, Na-Dene, Eskimos, and Amerindian populations (data were taken from the 11th and 12th International HLA Workshops [51,69]), obtained the genetic distances (DA) and the relatedness dendrogram, and performed the correspondence analysis. In particular, the Amerindian populations included in the present work belong to the following linguistics families: Arawakan (Wayúu and Terena Indians), Chibchan (Chimila, Wiwa, Arsario, Kogi, Arhuaco), Choco-Emberá (Jaidukama), Ge Pano Caribe (Xavantes, Mataco-Wichi, and Toba), Tupi-Guarani (Guarani), Barbacoan (Cayapa), Mayan (Mayans, Teenek), Mixe-Zoque (Mixe), Oto-Manguean (Mixtecans, Mazatecans, and Zapotecans), Uru-Chipaya (Uros), Uto-Aztecan (Nahuas and Mayos), and Andean groups, like Aymara, Quechuas, and Lamas [3,80,81].

Both the reference tables of the 11th and 12th International HLA Workshops [51,69] and www.allelefrequencies.net [35] (accessed 23 December 2024) were used in order to perform genetic comparisons among the populations tested. Genetic distances between populations (DA) [82] were calculated, and a Neighbor-Joining (NJ) relatedness dendrogram [83] was constructed from high-resolution HLA-DRB1 frequencies by using GNKDST and TreeView software (version 9.22.1), respectively [84,85].Both correspondence analysis in three dimensions and its bidimensional representation were carried out using the VISTA v5.05 computer program [86]. Correspondence analysis consists of a geometric technique that may be used for displaying a global view of the relationships among populations according to HLA (or other) allele frequencies. This methodology is based on the allelic frequency variance among populations (similar to the classical components methodology) and on a statistical visualization of the differences.

## 3. Results

### 3.1. Characteristic Chimila HLA Allelic and Extended Haplotype Frequencies

Twenty-one HLA-A and twenty-six HLA-B alleles were found in our Chimila sample studied in the present work. The most frequent ones were HLA-A*24:02 (42.50%), -A*68:01 (12.50%), and -A*24:03 (8.75%) and HLA-B*51:10 (38.75%), -B*35:43 (13.75%), and -B*78:02 (5.0%) (Table 2). Regarding class II alleles, seventeen different HLA-DRB1 and thirteen different HLA-DQB1 alleles were found, where HLA-DRB1*04:07 (51.25%), -DRB1*03:02 (7.5%), -DRB1*04:17 (7.5%), -DQB1*03:02 (50.0%), -DQB1*06:02 (11.25%), and -DQB1*03:01 (8.75%) were the most frequent ones (Table 2).

The most frequent Chimila A-B-DRB1-DQB1 haplotype found was HLA-A*24:02-B*51:10-DRB1*04:07-DQB1*03:02 (26.25%) (Table 2). This haplotype contains the most frequent alleles found in the studied Chimila population. It is quasi-specific of the Chimila population, as it occurs at a high frequency only in Chimila, although it is also found at very low frequencies in other Colombians and Panamanians [35]. The same occurs with the second most frequent haplotype (A*68:01-B*35:43-DRB1*04:07-DQB1*03:02; 6.25%) (Table 2), which is also frequently found in Chimila but is also present at lower frequencies in Colombians, Costa Ricans, and Panamanians [35]. The third (A*02:04-B*78:02-DRB1*04:17-DQB1*04:02; 5.0%), sixth (A*24:02-B*08:50-DRB1*03:01-DQB1*02:01; 2.5%), eighth (A*24:02-B*40:09-DRB1*03:02-DQB1*03:01; 2.5%),and ninth (A*24:03-B*38:01-DRB1*04:07-DQB1*06:03; 2.5%) most frequent haplotypes (Table 2) are specific to theChimila ethnicity, as they are not found in any other population worldwide [35]. The fourth (A*29:02-B*45:01-DRB1*15:01-DQB1*06:02; 3.75%) and fifth (A*02:01-B*15:01-DRB1*16:02-DQB1*03:01; 2.5%) most frequent haplotypes (Table 2) are found at relatively high frequencies in Chimila but also atlower frequencies in other populations like Colombian, Mexican, Brazilian, or Polish populations (at lower frequencies), among others [35]. The seventh most frequent haplotype (A*24:02-B*35:43-DRB1*04:07-DQB1*03:02; 2.5%) (Table 2) is also highly prevalentin the Wiwa population studied in the present work; it is also in Spanish and other Amerindian populations, like Nicaraguan, Colombian, Panamanian, or Costa Rican populations, at low frequencies [35].

### 3.2. Characteristic Wayúu HLA Allelic and Extended Haplotype Frequencies

Six HLA-A and thirteen HLA-B alleles were identified in theWayúu sample studied in the present work. The most frequent were HLA-A*02 (36.59%), -A*24 (34.15%), and -A*31 (14.63%) and HLA-B*15 (28.05%), -B*35 (18.29%), and -A*51 (15.85%) (Table 3). Ten different HLA-DRB1 and six different HLA-DQB1 alleles were also found in our Wayúu sample; HLA-DRB1*04:03 (35.37%), -DRB1*04:11 (20.73%), -DRB1*(13.41%), -DQB1*03:02 (60.98%), -DQB1*04:02 (19.51%), and -DQB1*03:01 (10.98%) were the most prevalent (Table 3).

The first most frequent haplotype found in our Wayúu sample (A*24-B*51-DRB1*04:03-DQB1*03:02; 9.59%) (Table 3) is also found in other Asian and Amerindian populations, like Indian, Japanese, Malayan, Sri Lankan, Colombian, Mexican, or Mayo populations, among others, but at lower frequencies than in Wayúu [35]. The second haplotype, A*02-B*15-DRB1*16:02-DQB1*03:01 (6.10%) (Table 3), is an Amerindian/South American haplotype also found at relatively high frequencies in Chimila and Mazatecan ethnicities, but also in Brazilians, Panamanians, Mexicans, and Colombians at low frequencies [35]. In the case of the third most frequent haplotype, A*68-B*15-DRB1*04:03-DQB1*03:02 (4.88%) (Table 3), it seems to have a mixed origin, being found in the Wiwa population and also in Indian, Malayan, Sri Lankan, German, and Polish populations but at low frequencies [35]. The fourth haplotype most frequently found in our Wayúu sample (A*02-B*51-DRB1*04:11-DQB1*04:02; 3.82%) (Table 3) seems to be quasi-specific of this population because it is frequently found in Wayúu and also only in Nicaraguan, Mexican, and USA Hispanic/Caribbean populations at very low frequencies [35]. The fifth one, A*02-B*35-DRB1*08:02-DQB1*04:02 (3.66%) (Table 3), has an Amerindian/Asiatic origin since it is found in Uros, Aymara, Quechua, and Guatemalan Mayan populationsat very high frequencies and at lower frequencies in Tarahumara, Otomi, Mexican Mayan, Mayo, Mexican mestizo, Colombian, Panamanian, Nicaraguan, Indian, Japanese, and Sri Lankan populations, among others [35]. The sixth most frequent Wayúu haplotype (A*24-B*15-DRB1*04:07-DQB1*03:02; 3.66%) (Table 3) is an Amerindian-specific haplotype frequently found in Wiwa, Mayo, and Guatemalan Mayans, and also in Uros, Otomi, Nicaraguans, Panamanians, Colombians, and Mexicans at lower frequencies [35]. The seventh haplotype found, A*24-B*35-DRB1*04:03-DQB1*03:02 (3.66%) (Table 3), is also found in other Amerindian populations, like Quechua, Aymara, Uros, Teenek, Wiwa, and Mexican Mayan, but also in some Asiatic (Indian, Chinese, Japanese, Vietnamese, or Malayan) and Caucasoid (Turkish German, Russian, or Polish) populations at very low frequencies [35]. The eighth most frequent haplotype found in our Wayúu sample (A*02-B*15-DRB1*04:11-DQB1*04:02; 3.49%) (Table 3) seems to be specific to the Wayúu population since it is also only found in a Brazilian sample at a frequency of 0.08% [35].

### 3.3. Characteristic Wiwa HLA Allelic and Haplotype Frequencies

After statistical calculations, twenty-one HLA-A, twenty-six HLA-B, fifteen HLA-DRB1, and twelve HLA-DQB1 different alleles were found in our Wiwa sample studied in the present work. The most frequent HLA class I alleles found were HLA-A*24:02 (44.9%), -A*02:01 (11.22%), and -A*68:01 (9.18%) and HLA-B*35:43 (37.76%), -B*15:01 (13.27%), and -B*40:02 (5.10%) (Table 3). Regarding HLA class II alleles, the most frequent ones were HLA-DRB1*04:07 (56.12%), -DRB1*08:02 (9.18%), and -DRB1*14:02 (8.16%) and HLA-DQB1*03:02 (65.31%), -DQB1*04:02 (8.16%), and -DQB1*02:02 (7.14%) (Table 4).

Regarding the HLA extended haplotypes obtained for the Wiwa sample studied in the present work, the most frequent haplotype (A*24:02-B*35:43-DRB1*04:07-DQB1*03:02; 21.43%) (Table 4) is also found in Chimila Amerindians and Colombians at relatively high frequencies but is also found in Costa Ricans, Nicaraguans, Panamanians, and Spanish peopleat low frequencies [35]. The second most frequent haplotype found, A*02:01-B*35:43-DRB1*04:07-DQB1*03:02 (6.12%) (Table 4), seems to be quasi-specific of the Wiwa population since it is also only found in other Amerindian populations, like Mexican Mayan, Nicaraguan, Colombian, or Panamanian populations, but at very low frequencies [35]. The same occurs with the third most frequent haplotype found, A*24:02-B*15:01-DRB1*04:07-DQB1*03:02 (5.10%) (Table 4), which is found at a relative high frequency in the Wiwa population but also at very low frequencies in other Amerindian populations, like Otomi, Colombian, Panamanian, Nicaraguan, and Mexican populations [35], so it may be assigned as quasi-specific of the Wiwa population. The fourth most frequent haplotype found, A*29:02-B*44:03-DRB1*07:01-DQB1*02:02 (4.08%) (Table 4), has a mixed background since it is also found in Spanish, Tunisian, Mexican, Canary Islander, Russian, and Brazilian populations, among others [35]. This is also the case of the seventh (A*24:02-B*07:02-DRB1*15:01-DQB1*06:02; 2.04%) (also found in Russian, Polish, Chinese, Brazilians, Sri Lankan, Indian, Spanish, German, or Colombian populations) and tenth (A*26:01-B*38:01-DRB1*13:01-DQB1*06:03; 2.04%) (found in Russian, Polish, Indian, Spanish, German, Portuguese, or Uros populations, among others) most frequent haplotypes found (Table 3) [35]. The fifth (A*68:01-B*15:01-DRB1*14:02-DQB1*03:02; 3.06%), sixth (A*02:01-B*35:43-DRB1*04:03-DQB1*03:02; 2.04%) and eighth (A*24:02-B*35:44-DRB1*04:07-DQB1*03:02; 2.04%) (Table 4) most frequent haplotypes are specific of the Wiwa population and not found in any other one. The ninth most frequent haplotype obtained, A*24:02-B*40:03-DRB1*04:07-DQB1*03:02 (2.04%) (Table 4), appears to be quasi-specific of the Wiwa population since it is also only found in the Mexican Xalapa population at lower frequencies [35].

### 3.4. Characteristic Barranquillan HLA Allelic and Haplotype Frequencies

Nineteen HLA-A and twenty-nine HLA-B different alleles were found, where HLA-A*02 (22.92%), -A* (18.75%), and -A*68 (8.04%) and HLA-B*35 (18.75%), -B*44 (9.82%), and -B*40 (8.04%) were the most frequent ones (Table 5). In the case of HLA class II, twenty-four HLA-DRB1 different alleles were found in our Barranquillan sample studied in the present work. The most frequent ones were HLA-DRB1*04:01 (19.35%), -DRB1*07:01 (10.12%), and -DRB1*15:01 (9.82%) (Table 5).

Regarding the HLA extended haplotypes obtained from our Barraquillan sample, the first most frequent haplotype found (A*24-B*35-DRB1*04:01; 4.51%) (Table 5) has a mixed background, but it is frequently found in Europeans/Mediterraneans, like Russian, Portuguese, Italian, Iranian Kurd, Iranian Yazd, Polish, or German populations, among others [35]. This is the same case of the second most frequent haplotype found in Barranquillans (A*29-B*44-DRB1*07:01; 2.38%) (Table 5), which is also frequently found in Israeli, Portuguese, Russian, Spanish, Polish, German, Otomi, or Brazilian populations, among others [35]. The third most frequent Barranquillan haplotype, A*33-B*14-DRB1*01:01 (2.08%) (Table 4), is also found in Mediterranean populations, like Iranian Azeris, Iranian Gorgan, Iranian Yazd, Spanish or Jewish populations, European populations, like Russians, Aleutians, or Germans, and some South American populations, like Wiwa, Chileans, Colombians, and Cubans [35]. The fourth most frequent haplotype found, A*02-B*51-DRB1*04:01 (1.79%) (Table 5), appears in a mixtureof populations, but it has a clear European/Mediterranean background, being frequently found in Portuguese, Polish, German, Spanish, English, and Jewish populations and in some Asian/American populations, like Japanese, Chinese, Indian, and Colombian, at lower frequencies [35]. The fifth most frequent haplotype found in Barranquillans, A*24-B*40-DRB1*03:02 (1.79%) (Table 5), appears to be quasi-specific of this population since it is only found in Barranquillan, Chimila, and Colombian populationsat relatively high frequencies [35]. It also occurs with the eleventh most frequent haplotype found in this sample (A*31-B*35-DRB1*03:02; 1.19%), also onlybeing found in Colombians and Nicaraguans at very low frequencies [35]. As in the case of the previous haplotypes described above, the sixth (A*02-B*39-DRB1*13:01; 1.49%) (found in Polish, Russian, Colombian, Bolivian, and South African populations, among others), the seventh (A*02-B*08-DRB1*03:01; 1.46%) (found in Russian, Madeiran, English, Irish, Iranian, Canary Island, Tanzanian, or Indian populations, among others), the eight (A*02-B*15-DRB1*04:01; 1.42%) (found in Russian, Swedish, Spanish, German, and South African populations, among others), the ninth (A*02-B*35-DRB1*04:01; 1.19%) (found in Gorgan, Russian, German, Polish, Panamanian, or Mexican populations, among others), and the tenth (A*02-B*44-DRB1*01:01; 1.19%) (found in Brazilian, Irish, English, Canary Island, German, or Russian populations, among others) most frequent haplotypes found in the Barranquillan sample of the present work show a mixed origin but with a clear European/Mediterranean background (Table 5) [35].

### 3.5. Genetic Distances (DA) Between the Chimila, Wiwa, Wayúu, and Barranquillan Populations Studied and Other Amerindian and Worldwide Populations

The genetic distances obtained in the present work between the Chimila, Wayúu, Wiwa, and Barranquillan populations and other Amerindian and worldwide populations included for comparison are shown in Supplementary Tables S1–S4.

The Chimila people are close to other Amerindians such as Mayo (Mexicans) (24.13 × 10^−2^), Mexican Mayans (27.10 × 10^−2^), or Teenek (Mexicans) (28.13 × 10^−2^) (Table S1). All other Amerindian populations included in the comparisons appear genetically close to the Chimila population (except for Tarahumara, Xavantes, Mataco-Wichi, and Guarani). Other Colombian ethnicities, such as Kogi (38.61 × 10^−2^), Jaidukama (42.11 × 10^−2^), Arsario (47.65 × 10^−2^), and Arhuaco (50.55 × 10^−2^), are also close to our Chimila population according to the DA genetic distances calculated in the present work (Table S1). The results also show that the Wiwa (28.28 × 10^−2^) and Wayúu (46.96 × 10^−2^) populations studied in the present work are genetically close to the Chimila population, which is not the case of the Barranquillan population, which is much further (60.18 × 10^−2^) (Table S1). Several of the genetically close Amerindian populations are distant in geography.

In the case of the Wayúu population, all Amerindian populations included in the genetic comparisons performed occupy the closest positions in the list, where Mapuche (22.22 × 10^−2^), Mixteco (26.06 × 10^−2^), Teenek (26.83 × 10^−2^), and Lamas (29.90 × 10^−2^) are the closest ones (Table S3). Other Colombian ethnicities, such as Kogi (36.50 × 10^−2^) or Arhuaco (38.15 × 10^−2^), also show low genetic distances with our Wayúu sample (Table S2). The Wiwa (43.44 × 10^−2^) and Chimila (46.96 × 10^−2^) populations studied in the present work are also close to the Wayúu population (Table S2). Again, the Barranquillans (88.72 × 10^−2^) appear very genetically far from the Wayúu and other Amerindian populations (Table S2). Several of the genetically close Amerindian populations are distant in geography.

The Wiwa sample included in the present study follows the same pattern as the Chimila and Wayúu samples. This Wiwa population shows the closest genetic distances to the other Amerindian populations of the other populations studied in the present work. Colombian ethnicities, like Kogi (15.27 × 10^−2^), Arhuaco (20.65 × 10^−2^), and Arsario (20.71 × 10^−2^), show some of the closest genetic distances to Wiwa (Table S3). Other Amerindian populations are also genetically very close to our Wiwa sample, such as the Mayan (12.20 × 10^−2^), Seri (15.79 × 10^−2^), Mayo (20.47 × 10^−2^), or Tarahumara (26.03 × 10^−2^) populations (Table S3). The Chimila (28.28 × 10^−2^) and Wayúu (43.44 × 10^−2^) samples in the present work also show very close genetic distances to the Wiwa sample, where Barranquillans (70.87 × 10^−2^) occupy a further position in the list (Table S3). Several of the genetically close Amerindian populations are distant in geography.

The genetic distances of the Barranquillans studied in the present work differ highly from the other Colombian-ethnicity genetic relationships presented in this study. In the case of Barranquillans, the genetically closest populations found are European populations, like Russian (17.93 × 10^−2^), Danish (18.18 × 10^−2^), French (18.92 × 10^−2^), or German (20.20 × 10^−2^) populations (Table S4). Some Mediterranean populations, like Italian (25.10 × 10^−2^), Basque (25.85 × 10^−2^), Spanish (27.06 × 10^−2^), Macedonian (32.20 × 10^−2^), Algerian (35.95 × 10^−2^), Moroccan (36.08 × 10^−2^), or Cretan (37.85 × 10^−2^) populations, are also very genetically close (Table S4). The Amerindian populations included in the comparisons appear genetically far from Barranquillans; the Quechua (54.62 × 10^−2^) population is the closest one, followed by the Chimila (60.18 × 10^−2^), Mapuche (63.59 × 10^−2^), and Mayan (66.60 × 10^−2^) populations (Table S4). The Wayúu and Wiwa populations studied in the present work are also situated very genetically far from the Barranquillan population, with a score of 88.72 × 10^−2^ and 70.87 × 10^−2^, respectively. Other Colombian ethnicities, such as Jaidukama (95.86 × 10^−2^) and Arsario (96.55 × 10^−2^), show the furthest genetic distances with Barranquillans (Table S4).

### 3.6. Neighbor-Joining Relatedness Dendrogram Construction and Correspondence Analysis by Using HLA-DRB1 Genetic Distances

The HLA-DRB1 relatedness dendrogram obtained following the Neighbor-Joining method (Figure 2) shows that the populations included in this work cluster together in two separate and different branches. In the upper one, Asiatic/Oceanian populations tend to cluster together in the same node, where Australian aborigines appear separated from the other ones, followed by Oceanians and continental Asiatic groups. In the same node, Caucasian (European/Mediterranean/North African) populations cluster together and are separated from the other ones present in this branch. As may be also inferred by the genetic distances, our Barranquillan sample appears within this Caucasian node. In the bottom node, a separated cluster of all the Amerindian populations stands out, including our Chimila, Wayúu, and Wiwa samples studied in the present work (Figure 2).

Similar to what occurs in the relatedness dendrogram described above, the correspondence analysis performed shows that the Chimila, Wayúu, and Wiwa populations are placed within the Amerindians group (Figure 3, red). On the other hand, the Barranquillan population clusters together with the Caucasian (European/Mediterranean) populations included in the comparisons performed in this work. The data extracted from the correspondence analysis results are coincidental with those obtained in the genetic distances calculations and relatedness phylogenetic NJ tree construction.

## 4. Discussion

### 4.1. Genetics of Urban Barranquillans Shaped by Centuries of European and Mediterranean Admixture

Barranquilla has been, since its foundation in 1813, a key city in the population movements of Colombia, serving as the country’s main Caribbean port and one of the most significant in the region [28]. Spaniards, as the initial colonizers, established settlements and mixed with the local population [87]. Later, during the 19th and 20th centuries, waves of immigrants arrived from Europe and the Middle East, especially from the Mediterranean Levant [87]. Most of the immigration to Colombia entered through the Barranquilla Port, considerably increasing its population and making it one of the most cosmopolitan cities in the country [87]. Treaties were signed between the cities of Lübeck, Bremen, and Hamburg, on the one side, and New Granada (Colombia), on the other, in 1854 [88].

Around the same time, large numbers of Italian families migrated to Barranquilla to establish agricultural colonies, although later revolts led to the rupture of diplomatic relations between the two countries [89]. Throughout the 20th century, the city of Barranquilla received numerous immigrant families from different European countries, such as Slovenia [90], Spain [91], Italy [89], or the Netherlands [92], who settled there, forming family nuclei and, consequently, increasing the genetic diversity of the city’s population.

Barranquilla has also been an immigration center for people from the Middle East and Asia [93]. Migration waves included people from countries such as Lebanon [94], India [95], or Japan [96], greatly influencing the culture of the region and, consequently, the genetic landscape, establishing important communities of these nationalities in the city.

As a result of this admixture of cultures and people from other parts of the world, the inhabitants of Barranquilla, descendants of these diverse immigrants, exhibitvery varied and diverse HLA genetics. As is shown in this study, the frequency of the most frequent extended HLA haplotypes in Barranquillans is very homogeneous (Table 4), with no single haplotype dominating, indicative of abundant genetic admixtureand lower levels of population isolation. In contrast, more isolated Amerindian populations, such as the Chimila, Wayúu, or Wiwa populations studied in the present work, do present very frequent characteristic haplotypes (Table 1, Table 2 and Table 3). While in the Barranquilla population, the most frequent HLA haplotype has a frequency of 4.51%, the Chimila, Wayúu, and Wiwa populations show frequencies of 26.26%, 9.49%, and 21.43%, respectively, indicating greater homogeneity and singularity of these populations compared to that of Barranquilla.

### 4.2. Amerindian Uniqueness Supported by Chimila, Wiwa, and Wayúu HLA Genetics

**Figure 4:**
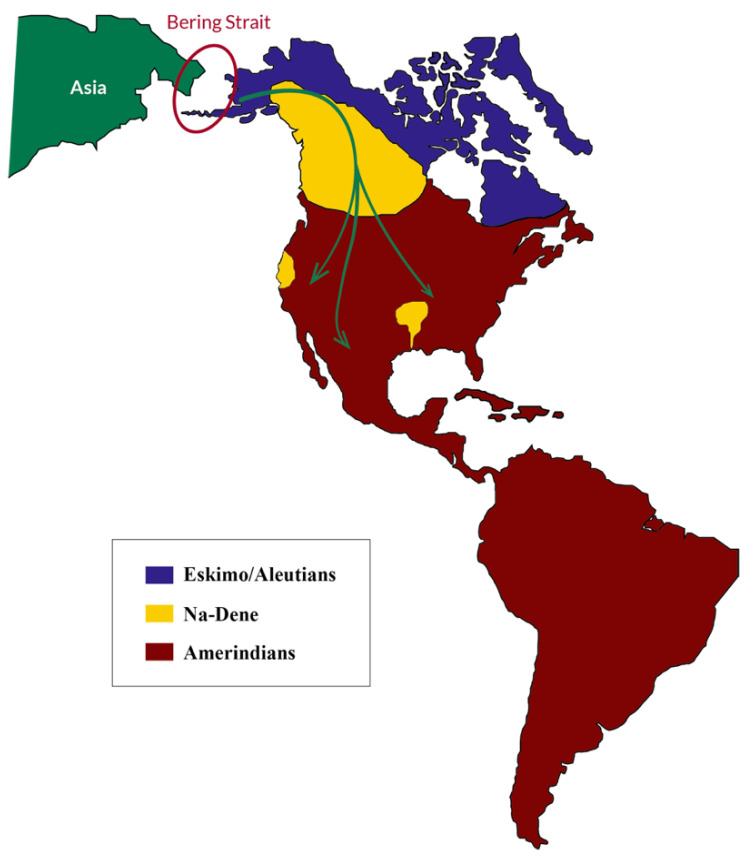
Map showing population movements from Asia through the Bering Strait for the American population according to the Clovis theory. Different population groups crossed the land bridge during the last ice age and gave rise to the three ancestral ethnicities of America: Aleutians, Na-Dene, and Amerindians. This is the classic theory that is now plainly dismissed because most ancient human settlements are found in Chile and California (see the Discussion and Figure 5) [113,114,115].

The results obtained in this and previous studies on the HLA genetics of Amerindian groups in Colombia support many previous studies that demonstrate the genetic uniqueness of Amerindians and their descendant populations [55,67,97,98]. As shown in Figure 3 and Figure 4, Amerindians form a separate genetic cluster without influences from surrounding populations such as Asians or Na-Dene/Eskimo. The genetic homogeneity in American Indians, as demonstrated by HLA studies, is further supported by studies based on mitochondrial inheritance: Amerindians are grouped into only five mitochondrial haplogroups (A, B, C, D, and X), showing a certain genetic homogeneity in the maternal lineage in American Indians [99,100]. Haplogroup X is even more unique because it is quasi-specific to the indigenous populations of North America, although it may also be found in western Eurasia. This haplogroup is particularly frequent in European regions, where the X2j lineage is closely related to the American X2a [101]. This genetic evidence supports the debated Solutrean hypothesis, which suggests that some of the first inhabitants of America came from Europe across the Atlantic carrying haplogroup X and other cultural traits, such as Solutrean-type stone tool fabrication techniques from North Iberia and southern France. However, it is also possible that cultural and gene flow could also occur from America to Europe [102,103].

On the other hand, patrilineal genetics (Y chromosome) further support the distinctiveness and homogeneity in Amerindian populations. The most frequent Y-chromosome haplogroup across America is haplogroup Q, a pan-American haplogroup that represents all Native American lineages, at least from Mesoamerica and South America [104,105]. The most common sub-haplogroups are Q-M848 and Q-Z780 (which occurs at low frequency) [104,106,107]. A recent phylogenetic study and analysis of Y-chromosome SNPs in Amerindian groups [104] suggests that the Q-Z780 sub-haplogroup may have appeared in the Americas around 19,300 years BP, challenging the Clovis-first model and supporting an earlier human settlement of the continent [104].

The data provided by the present (and other) study on the HLA genetic and haplotype profile of the Chimila, Wayúu, and Wiwa ethnic groups of Colombia corroborate that Amerindians form an isolated and singular genetic group with unique characteristics (see Conclusions). Additional studies using different genetic markers further confirm the Amerindian genetic singularity [53,67,100,104,105,106,107,108]. Autosomic markers other than HLA do not contradict these results and are not detailed in this paper.

### 4.3. HLA Genetics of Chimila, Wayúu, and Wiwa Amerindians Support Trans-Pacific Contacts for American Populations

One of the most debated theories on the American population established that several oceanic freezing events in the Beringia took place between 40,000 and 10,500 years BP [109], and that this fact, together with the drop in the sea level (around 120 m), allowed different population groups to move from Asia to North America through a “land bridge”. During this ice age, the present-day Canadian territory would have been fully covered by two immense ice plates, but a non-frozen corridor would have been formed around 14,000 years BP, facilitating the movement from Asia towards the south [110,111,112]. This led to the Three Waves theory during the 20th century. It stated that three different population movements towards America took place between 13,500 and 14,000 years BP [113,114], giving rise to the ancestor lineages of all present-day main American ethnic groups: Eskimos, Na-Dene, and Amerindians [114,115] (Figure 4). Also, the Clovis culture was considered the most ancient culture of the First American Inhabitants during the 20th century [116].

**Figure 5:**
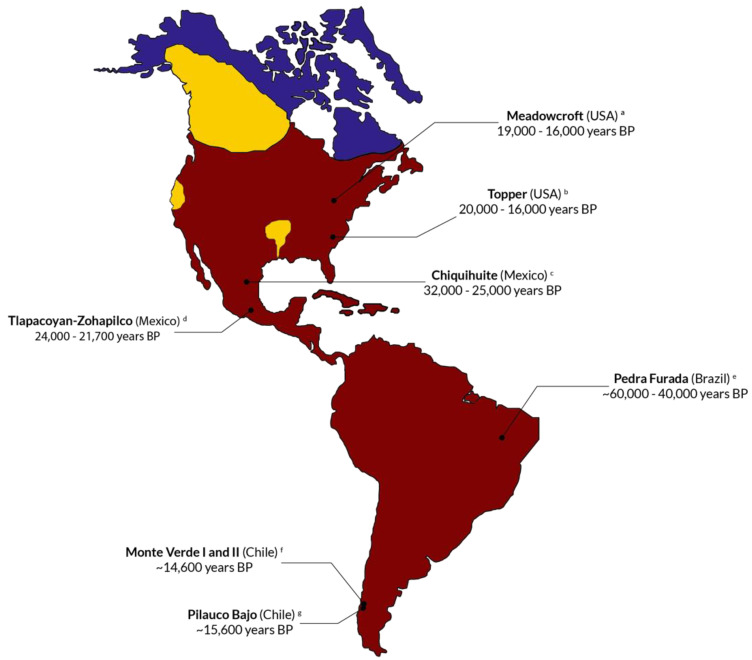
Map showing pre-Clovis human settlements across the American continent. These first settlements have been hotly debated, but robust dating procedures have been carried out, and the conclusions are now firm. See references ^a^ [116], ^b^ [117], ^c^ [118], ^d^ [119], ^e^ [120], ^f^ [121,122], and ^g^ [123] for more information about the settlements shown.

However, many recently found human settlements throughout the American continent (Figure 5) contradict the Clovis theory and leave the door open for previous and different movements from Asia or other places to America.

The Chimila, Wayúu, and Wiwa samples studied in the present work revealed the presence of HLA-A*24 (A*24:02) and -B*48 alleles in their genetic profile at varying frequencies. Noteworthily, these alleles are characteristic of populations inhabiting Southeast Asia and the Asian and Pacific islands; they are found in Polynesian and Easter Islander genetic profiles, even in some sites located over 4000 km from the Pacific coast of South America [35,124,125,126]. The distribution of these particular alleles cannot be fully explained by the three-wave model postulated by Greenberg [114,115] for the first American population, which suggests that the first migrations to the Americas occurred solely through Beringia. If these alleles had been introduced via the Beringian migration route, they would likely be more evenly distributed across the continent rather than being concentrated along or near the Pacific coastline. This suggests the existence of direct genetic and population exchanges between Pacific Islanders and the indigenous peoples of the Americas, though it remains unclear whether these interactions were bidirectional or one-way. In 1947, the Norwegian explorer Thor Heyerdahl demonstrated, with his Kon-Tiki expedition, that a transpacific trip between the Port of Callao, in Peru, and Easter Island could be carried out by using only a rudimentary boat as a means of transport (a “totora” or reed-made boat), like the one that could have been used in prehistoric times in the America highlands (e.g., Titikaka Lake). Infact, Easter Island’s giant statues are very similar to those found in Tiwanaku culture at Titikaka Lake (5000 m above sea level; Bolivia/Peru) [126,127]. This resemblance suggests that ancient trans-Pacific voyages could have occurred, possibly linking indigenous American and Oceanian populations. All of the above could explain why the HLA-A*24(:02) and -B*48 alleles are in the Amerindian populations studied in the present work and also in others distributed along or close to the Pacific coast of America, such as Quechua (Peru and Bolivia) [52], Aymara (Peru and Bolivia) [50], Mayo (Mexico) [41], and Mapuche (Chile) [64], among others. This distribution supports the hypothesis that the settlement of America did not occur only from Asia (through the Bering Strait) but that the peoples of the continent had Pacific transoceanic contacts with other populations.

In addition to this genetic evidence, numerous archaeological findings support population contact between the Pacific Islandersand the inhabitants of the west coast of South America. The sweet potato (*Ipomoea batatas*) is an edible root endemic to South America that is also frequently bred and eaten in Polynesia. It was believed that the Spanish conquerors took it to the Pacific islands after the conquest of America, but traces of the existence of this root have been found on Mangaia Island, the Cook Islands archipelago, dating back to 1000 AD [128,129], long before the arrival of the Spanish and Portuguese to America. This would indicate a probable contact between the inhabitants of the continent and those of the Pacific islands. Other archaeological evidence of these contacts relates to skulls found in Isla Mocha and Tunquén, Chile [130,131]. These skulls have a typical Polynesian morphology with a marked pentagonal shape [130], which may indicate the arrival of Pacific inhabitants to America in pre-Columbian times, establishing interactions between them and Mapuches.

These facts together, along with the data obtained in the present work on the Chimila, Wayúu, and Wiwa ancestral ethnicities of Colombia, support the existence of bidirectional trans-Pacific contacts between Pacific island inhabitants and Amerindians in prehistoric times that influenced the American population. The continent was complexly populated for thousand years, not only through the Bering Strait but also through transoceanic contacts with Oceanians and even Europeans [106,107].

## 5. Conclusions and the First American Population

### 5.1. American Population [30,31,131]

- Africans were brought to Colombia as slaves by the Spaniards [30,31]. They were excluded from this analysis because they are HLA genetically very similar to Mediterraneans [131] and thus could hinder this study’s calculations and conclusions.
- After Columbus’s arrival in 1492 AD, the Amerindian population from Alaska to South America (about 80 million) was drastically reduced by 1552 AD (8 million) because of new European-borne diseases (mainly influenza, smallpox, and measles) and war [108,132]. This drastic population reduction likely caused a genetic bottleneck, which explains why modern Amerindian HLA profiles do not always follow strict geographic patterns. The loss of genetic diversity may be attributed to the selective survival of certain alleles in populations able to present peptides derived from newly introduced pathogens.

### 5.2. Conclusions

The peopling of America was a complex and multifactorial process that certainly did not occur in prehistory by the entry of people (and genes) through the Bering Strait only. The arrival of people from Oceania through the Pacific Ocean occurred, probably with bidirectional contacts. More studies are ongoing, and the prehistoric gene flow through the Atlantic Ocean with Europeans cannot be discarded.

The Amerindian HLA genetic profile is different from the rest of the world. This may be due to isolation and/or to the bottleneck that occurred after 1492 AD and the massive entrance of Europeans.

This and other studies in the Chimila, Wayúu, and Wiwa [35,133,134,135] and Barranquillan [35,136] populations support the genetic uniqueness of Amerindian populations and highlight the possible different ways of the American population.

## Figures and Tables

**Table 2:** HLA-A-B-DRB1-DQB1 extended haplotype frequencies found in the Chimila sample studied in the present work (*n* = 46).

Haplotype	Freq. (%)	Origin [35]
A*24:02-B*51:10-DRB1*04:07-DQB1*03:02	26.25	Chimila
A*68:01-B*35:43-DRB1*04:07-DQB1*03:02	6.25	Chimila
A*02:04-B*78:02-DRB1*04:17-DQB1*04:02	5.00	Chimila
A*29:02-B*45:01-DRB1*15:01-DQB1*06:02	3.75	Amerindian/Mixed
A*02:01-B*15:01-DRB1*16:02-DQB1*03:01	2.50	Amerindian
A*24:02-B*08:50-DRB1*03:01-DQB1*02:01	2.50	Chimila
A*24:02-B*35:43-DRB1*04:07-DQB1*03:02	2.50	Amerindian
A*24:02-B*40:09-DRB1*03:02-DQB1*03:01	2.50	Chimila
A*24:03-B*38:01-DRB1*04:07-DQB1*06:03	2.50	Chimila

**Table 3:** HLA-A-B-DRB1-DQB1 extended haplotype frequencies found in the Wayúu sample studied in the present work (*n* = 46).

Haplotype	Freq. (%)	Origin [35]
A*24-B*51-DRB1*04:03-DQB1*03:02	9.59	Amerindian/Asiatic
A*02-B*15-DRB1*16:02-DQB1*03:01	6.10	Amerindian
A*68-B*15-DRB1*04:03-DQB1*03:02	4.88	Mixed
A*02-B*51-DRB1*04:11-DQB1*04:02	3.82	Specific
A*02-B*35-DRB1*08:02-DQB1*04:02	3.66	Amerindian/Asiatic
A*24-B*15-DRB1*04:07-DQB1*03:02	3.66	Amerindian
A*24-B*35-DRB1*04:03-DQB1*03:02	3.66	Amerindian/Mixed
A*02-B*15-DRB1*04:11-DQB1*04:02	3.49	Specific

**Table 4:** HLA-A-B-DRB1-DQB1 extended haplotype frequencies found in the Wiwa sample studied in the present work (*n* = 46).

Haplotype	Freq. (%)	Origin [35]
A*24:02-B*35:43-DRB1*04:07-DQB1*03:02	21.43	Amerindian
A*02:01-B*35:43-DRB1*04:07-DQB1*03:02	6.12	Amerindian
A*24:02-B*15:01-DRB1*04:07-DQB1*03:02	5.10	Amerindian
A*29:02-B*44:03-DRB1*07:01-DQB1*02:02	4.08	Mixed
A*68:01-B*15:01-DRB1*14:02-DQB1*03:02	3.06	Wiwa
A*02:01-B*35:43-DRB1*04:03-DQB1*03:02	2.04	Mixed
A*24:02-B*07:02-DRB1*15:01-DQB1*06:02	2.04	Wiwa
A*24:02-B*35:44-DRB1*04:07-DQB1*03:02	2.04	Wiwa
A*24:02-B*40:03-DRB1*04:07-DQB1*03:02	2.04	Wiwa
A*26:01-B*38:01-DRB1*13:01-DQB1*06:03	2.04	Mixed

**Table 5:** HLA-A-B-DRB1 extended haplotype frequencies found in the Barranquillan sample studied in the present work (*n* = 168).

Haplotype	Freq. (%)	Origin [35]
A*24-B*35-DRB1*04:01	4.51	European/Mediterranean
A*29-B*44-DRB1*07:01	2.38	European/Mediterranean
A*33-B*14-DRB1*01:01	2.08	European/Mediterranean
A*02-B*51-DRB1*04:01	1.78	European/Mediterranean
A*24-B*40-DRB1*03:02	1.78	Barranquillan
A*02-B*39-DRB1*13:01	1.49	European/Mediterranean
A*02-B*08-DRB1*03:01	1.45	European/Mediterranean
A*02-B*15-DRB1*04:01	1.42	European/Mediterranean
A*02-B*35-DRB1*04:01	1.19	European/Mediterranean
A*02-B*44-DRB1*01:01	1.19	European/Mediterranean
A*31-B*35-DRB1*03:02	1.19	Barranquillan

## Data Availability

Genetic raw data about the HLA profiles of the Chimila, Wayúu, Wiwa, and Barranquillan cohorts obtained in the present work are available under reasonable request to the corresponding authors in the three years following the publication of this article.

## Supplementary Materials

The following supporting information can be downloaded at https://www.mdpi.com/article/10.3390/genes16030286/s1: Tables S1–S4 showing the genetic distances(DA) of the Colombian populations studied in the present paper when compared to worldwide populations.
