# Supplementary material for: The Origin of Amerindians: A Case Study of Secluded Colombian Chimila, Wiwa, and Wayúu Ethnic Groups and Their Trans-Pacific Gene Flow
Source: Genes (Basel). 2025 Feb 27;16(3):286. doi: 10.3390/genes16030286 (PMC11942480; doi:10.3390/genes16030286)
Supplement: Supplementary file 1 [file genes-16-00286-s001.zip › genes-3391314-supplementary.pdf]

Table S1. Genetic distances (DA) calculated from high-resolution HLA-DRB1 frequencies of Chimila sample studied in present work and all other world-wide populations included in the comparisons.

Population	DA ($x10^{-2}$)	Population	DA ($x10^{-2}$)
Mayos	24.13	Algerians	64.82
Mexican Mayans	27.10	Xavantes	64.85
Teenek	28.13	Aleuts	65.87
Wiwa	28.28	Spanish Basques	66.18
Lakota Sioux	31.86	Chuvashians	66.22
Mazatecans	32.14	Macedonians	66.62
Nahuas	38.26	Khoton Mongolians	66.71
Kogi	38.61	Mataco Wichi	67.75
Mixteco	39.88	Khalk Mongolians	70.36
Jaidukama	42.11	Guarani	70.77
Cayapa	42.67	Moroccan Jews	70.98
Toba Pilaga	45.73	Cretans	72.46
Lamas	46.89	Lebanese-NS	73.45
Wayúu	46.96	Singapore Chinese	73.65
Seri	46.99	Buyi	73.85
Quechuas	47.16	Japanese	74.35
Arsario	47.65	Manchu	76.45
Eastern Toba	50.00	Sardinians	77.64
Arhuaco	50.55	Fidji	79.33
Mixe	50.95	Athabaskans	80.43
Zapotecans	51.54	New Caledonians	80.46
Mapuche	54.86	Mandang	81.06
French	55.16	Papua New Guineans	81.59
Russians	56.09	Kets	81.62
Albanians	56.93	Tlinglit	84.18
Danish	57.62	Western Samoans	86.45
Terena	58.22	Evenks	86.94

Moroccans	58.66	Ainu	88.80
Germans	58.91	Chukchi	89.05
Uros	59.23	Rabaul	89.13
Tarahumaras	59.95	Nivkhs	92.14
Barranquilla	60.18	Eskimos	93.32
Spaniards	60.88	Udegeys	95.26
Tuvinians	61.18	Koryaks	95.26
Aymaras	62.39	Cape-York	96.50
Italians	62.82	Central-Desert	97.30
Koreans	63.87	Kimberley	98.78
Lebanese-KZ	64.57	Yuendumu	100.00

Table S2. Genetic distances (DA) calculated from high-resolution HLA-DRB1 frequencies of Wayúu sample studied in present work and all other world-wide populations included in the comparisons.

Population	DA ($x10^{-2}$)	Population	DA ($x10^{-2}$)
Mapuche	22.22	Chukchi	66.42
Mixteco	26.06	Albanians	66.70
Teenek	26.83	Koreans	66.87
Lamas	29.90	Khalk Mongolians	67.22
Zapotecans	31.76	Eskimos	68.22
Lakota Sioux	32.51	Tlinglit	68.35
Mixe	34.90	Tuvinians	69.18
Mayans	35.01	Koryaks	69.61
Mazatecans	36.36	Macedonians	69.97
Kogi	36.50	Ainu	70.88
Guarani	36.74	New Caledonians	71.20
Mayos	36.77	Mandang	71.40
Uros	37.60	Moroccans	71.90
Arhuaco	38.15	Fidji	72.02
Eastern Toba	38.49	Aleuts	72.14
Nahuas	39.35	Lebanese-NS	73.04
Wiwa	43.44	Manchu	73.88
Quechuas	43.66	Spaniards	74.53
Cayapa	46.50	Italians	74.89
Chimila	46.96	Moroccan Jews	75.19
Aymaras	48.71	Cretans	75.27
Tarahumaras	49.64	Khoton Mongolians	76.09
Xavantes	50.49	Chuvashians	78.01
Terena	50.50	Algerians	78.04
Arsario	51.66	Spanish Basques	78.13
Mataco Wichi	53.79	Germans	78.40
Toba Pilaga	55.79	Papua New Guineans	79.73

Seri	58.27	Evenks	83.18
Jaidukama	58.74	Danish	83.62
Lebanese-KZ	59.19	Sardinians	83.70
Athabaskans	62.95	Udegeys	85.85
Buyi	62.96	Barranquilla	88.72
Rabaul	63.68	Cape-York	89.33
French	64.40	Nivkhs	93.11
Western Samoans	65.68	Central-Desert	93.38
Singapore Chinese	65.68	Kimberley	93.54
Japanese	66.20	Yuendumu	94.50
Russians	66.22	Kets	95.87

Table S3. Genetic distances (DA) calculated from high-resolution HLA-DRB1 frequencies of Wiwa sample studied in present work and all other world-wide populations included in the comparisons.

Population	DA (x10^{-2})	Population	DA (x10^{-2})
Mayans	12.20	Moroccans	60.15
Kogi	15.27	Japanese	60.58
Seri	15.79	Chuvashians	61.33
Mayos	20.47	Spanish Basques	61.65
Arhuaco	20.65	Italians	62.21
Arsario	20.71	Udegeys	62.53
Tarahumaras	26.03	Aleuts	63.28
Chimila	28.28	Lebanese-KZ	63.65
Uros	28.46	Algerians	64.46
Quechuas	28.52	Albanians	65.26
Lakota Sioux	30.99	Cretans	66.13
Cayapa	31.43	Manchu	66.26
Teenek	31.50	Chukchi	66.65
Aymaras	33.81	Moroccan Jews	67.34
Mixteco	34.53	Koryaks	67.35
Nahuas	35.78	Nivkhs	68.37
Lamas	36.21	Western Samoans	69.65
Mazatecans	37.75	Eskimos	69.84
Zapotecans	38.89	Macedonians	70.32
Mapuche	39.84	Sardinians	70.60
Eastern Toba	40.06	Barranquilla	70.87
Jaidukama	41.83	Khoton Mongolians	71.24
Toba Pilaga	42.48	Singapore Chinese	74.07
Mixe	42.69	Ainu	74.18
Wayúu	43.44	Lebanese-NS	75.50
Athabaskans	46.10	Buyi	78.05
Xavantes	48.20	Fidji	80.75
Tuvinians	50.66	New Caledonians	80.81

Tlinglit	53.45	Kets	81.07
Terena	53.85	Guarani	81.15
Spaniards	55.01	Evenks	84.19
Koreans	56.79	Papua New Guineans	86.43
Germans	56.85	Cape-York	86.88
French	57.53	Rabaul	89.54
Danish	58.52	Mandang	90.70
Russians	58.58	Central-Desert	91.45
Mataco Wichi	58.90	Yuendumu	94.23
Khalk Mongolians	59.63	Kimberley	97.04

Table S4. Genetic distances (DA) calculated from high-resolution HLA-DRB1 frequencies of Barranquillan sample studied in present work and all other world-wide populations included in the comparisons.

Population	DA (x10^{-2})	Population	DA (x10^{-2})
Russians	17.93	Udegeys	62.76
Danish	18.18	Mapuche	63.59
French	18.92	Athabaskans	64.80
Germans	20.20	Mayans	66.60
Aleuts	20.27	Toba Pilaga	68.61
Chuvashians	21.12	Nivkhs	69.11
Tuvinians	24.81	Mandang	69.11
Italians	25.10	Wiwa	70.87
Spanish Basques	25.85	Nahuas	71.86
Spaniards	27.06	Papua New Guineans	74.25
Khalk Mongolians	31.18	Tarahumaras	74.26
Macedonians	32.20	Arhuaco	74.52
Khoton Mongolians	35.81	Uros	74.84
Algerians	35.95	Ainu	75.72
Moroccans	36.08	Eastern Toba	76.19
Albanians	36.60	Rabaul	78.93
Cretans	37.85	Lamas	79.93
Koreans	40.92	Aymaras	80.75
Manchu	42.12	Mayos	80.83
Moroccan Jews	42.30	Mataco Wichi	81.57
Evenks	44.63	Cayapa	84.42
Sardinians	45.01	Mazatecans	86.27
Kets	45.80	Seri	87.27
Chukchi	46.62	Wayúu	88.72
Lebanese-NS	48.69	Mixteco	89.35
Lebanese-KZ	48.76	Central-Desert	90.88
New Caledonians	49.79	Zapotecans	91.72

Japanese	52.84	Terena	92.19
Tlinglit	53.25	Cape-York	92.59
Quechuas	54.62	Yuendumu	92.60
Singapore Chinese	54.71	Kogi	93.42
Buyi	55.98	Guarani	94.26
Western Samoans	58.22	Mixe	94.80
Eskimos	59.42	Teenek	94.83
Fidji	59.66	Xavantes	95.36
Chimila	60.18	Kimberley	95.83
Koryaks	62.10	Jaidukama	95.86
Lakota Sioux	62.45	Arsario	96.55
